# Diagnostic potential of miR-200 family members in gingival crevicular fluid for chronic periodontitis: correlation with clinical parameters and therapeutic implications

**DOI:** 10.1186/s12903-023-03174-w

**Published:** 2023-07-31

**Authors:** Shi-Lei Yu

**Affiliations:** HangZhou Dental Hospital, HangZhou, 310000 Zhejiang China

**Keywords:** Chronic periodontitis, miR-200 family, Gingival crevicular fluid (GCF), Diagnostic biomarkers, Early detection and management

## Abstract

**Objective:**

The purpose of this study was to evaluate the potential of miR-200 family members in gingival crevicular fluid (GCF) as diagnostic biomarkers for chronic periodontitis (CP), aiming to provide valuable insights for the early detection and management of the disease.

**Methods:**

GSE89081 dataset profiled miRNAs in GCF derived from 5 healthy and 5 periodontitis was analyzed by GEO2R. Quantitative real-time PCR was used to quantify the expression levels of miR-200 family members (miR-200a-3p, miR-200a-5p, miR-200b-3p, miR-200b-5p, miR-200c-3p, miR-200c-5p, miR-141-3p, miR-141-5p, and miR-429) in the GCF samples from 103 CP patients and 113 healthy controls. Receiver operating characteristic (ROC) curve analysis was used to evaluate the diagnostic potential of miR-200 family members in differentiating CP patients from healthy controls.

**Results:**

By analyzing the GSE89081 dataset, miR-200a-5p, miR-200b-5p and miR-200c-5p were significantly upregulated in GCF of the CP patients compared to the healthy control. In this study, miR-200a-3p, miR-200a-5p, miR-200b-3p, miR-200b-5p, miR-200c-3p, miR-200c-5p were significantly increased in GCF of CP patients compared to the healthy control, while miR-141 and miR-429 did not show significant differences. MiR-200a, -200b and 200c had good diagnostic value, and when these miRNAs were combined, they demonstrated excellent diagnostic value for CP with an AUC of 0.997, sensitivity of 99.03%, and specificity of 98.23%. MiR-200a, -200b and 200c in GCF showed significant and positive correlation with plaque index (PI), gingival index (GI), bleeding on probing (BOP), clinical attachment level (CAL), and probing pocket depth (PPD).

**Conclusion:**

MiR-200a, -200b and 200c in GCF may serve as potential biomarkers for the early diagnosis of CP, which was correlated with clinical parameters, being therapeutic targets for CP.

## Introduction

Chronic periodontitis (CP) is a prevalent chronic inflammatory disease of the periodontium, which is caused by the interaction between the host immune response and a microbial biofilm on the tooth surface [1, 2]. It is characterized by the progressive destruction of the periodontal tissues, including the gingiva, periodontal ligament, and alveolar bone [3]. CP is highly prevalent, affecting more than half of adults in China, Europe, and the United States, with ranging from 70 to 90% among individuals aged 60 to 74 [4]. Moreover, CP has also been associated with systemic diseases, such as cardiovascular diseases (CVD), diabetes, and adverse pregnancy outcomes [5, 6]. The etiopathogenesis of CP is believed to be multifactorial, with host genetic, environmental, and microbiological factors being the three major parameters that can determine the natural history of the disease [7]. Gingival crevicular fluid (GCF), a physiological fluid and an inflammatory exudate, is derived from the gingival plexus of blood vessels in the gingival corium beneath the epithelium lining of the dentogingival space and its existence has been recognized since the nineteenth century [8].

MicroRNAs (miRNAs) have emerged as important regulators in various biological processes, including cell proliferation, differentiation, apoptosis, and inflammation [9]. Dysregulation of miRNAs has been implicated in CP, with altered expression detected in the GCF of affected individuals [10]. For example, miR-3198 in GCF was associated with periodontitis and demonstrated good diagnostic ability (AUC = 0.72), while a combination of miR-3198 and miR-4299 in GCF showed an AUC value of 0.86 with a sensitivity of 68% and specificity of 96% [11]. Additionally, another study showed that miR-1226 in GCF may serve as a promising biomarker for periodontal disease, providing additional information to commonly used clinical parameters for diagnosis and prognosis of the disease [12]. Monitoring the abnormal expression of miRNAs in GCF could thus serve as a valuable tool for diagnosing and prognosing CP by targeting critical biological processes involved in the disease’s pathogenesis.

The miR-200 family, consisting of miR-141, miR-200a, miR-200b, miR-200c, and miR-429 [13], has been implicated in various inflammatory diseases such as inflammatory bowel diseases [13, 14] and nonalcoholic steatohepatitis [15]. Recent advancements in dental technology and the application of artificial intelligence, particularly convolutional neural networks, have facilitated the development of automated tooth segmentation approaches using 3D cone-beam computed tomography images, addressing limitations related to root anatomy, scattering, immature teeth, metal artifacts, and time consumption [16]. Machine learning algorithms, including artificial intelligence, have demonstrated promise in analyzing large-scale biological data, including miRNA expression profiles, to identify potential biomarkers and comprehend disease mechanisms [17, 18]. By harnessing the computational power of machine learning, researchers can uncover hidden patterns, identify potential biomarkers, and gain deeper insights into periodontitis mechanisms [19]. through an analysis of the GSE89081 dataset, which comprises miRNA profiles in GCF of CP patients using GEO2R, the upregulation of miR-200a-5p, miR-200b-5p, and miR-200c-5p was revealed in the GCF of CP patients.

Therefore, the rationale for this study is to investigate the expression levels of miR-200 family members in the GCF of CP patients and explore their correlation with clinical parameters. The objective of the study is to provide insights into the potential role of miR-200 family members in the pathogenesis of CP and assess their clinical significance as biomarkers for diagnosing and prognosing the disease.

## Materials and methods

### Identification of gene expression data in CP patients

GSE89081 (Platform: GPL22600) was downloaded from GEO (https://www.ncbi.nlm.nih.gov/geo/query/acc.cgi?acc=GSE89081), which profiled miRNAs in GCF derived from ten subjects (5 healthy and 5 periodontitis) using miRCURY LNA™ Universal RT microRNA PCR System. The data was analyzed by GEO2R.

### Study population

A total of 216 participants were recruited for this study between January 2021and January 2023, including CP group (103 systemically healthy patients with CP) and control group [113 systemically healthy participants without systemic disease and no sites showing probing depth (PD) ≥ 4 mm, clinical attachment level (CAL) ≥ 4 mm, or radiographic signs of bone loss [20]. The diagnosis and staging of CP were based on the *Clinical Application of the New Classification of Periodontal Diseases* [21]. The CP patients were selected based on specific criteria [22, 23]: which included: (1) a minimum of 40% of sites with CAL ≥ 2 mm and PD ≥ 4 mm; (2) at least one site in each quadrant with crestal alveolar bone loss ≥ 2 mm confirmed by digital periapical radiographs; and (3) at least 40% of sites with bleeding on probing (BOP). Exclusion criteria were applied, including: (1) use of antibiotics, immunosuppressive drugs, or anti-inflammatory drugs in the past three months; (2) periodontal therapy in the past three months; (3) diabetic, immunocompromised, pregnant or lactating patients; (4) oral cavity cancer; (5) history of excessive drinking; and (6) allergy to local anesthetics. All participants provided written informed consent before participating in the study, which was approved by the Ethics Committee of the hospital.

### GCF sampling

Prior to collecting GCF samples using periopaper strips, it is essential to ensure that the oral cavity is properly prepared to minimize contamination and ensure reliable results. To achieve this, all participants are typically required to rinse their mouth with sterile water and an oral antiseptic solution before the collection process begins. Periopaper strips are a small, absorbent paper that allows for the non-invasive collection of GCF samples, making it a convenient and reliable method for monitoring oral health [24]. To collect the sample, the strip should be placed in the crevice between the gum and the tooth to ensure optimal contact with the GCF fluid. After 30 s, thde strip should be carefully removed and placed in a transport vial containing a stabilizing solution to preserve the integrity of the sample. Samples (10 µl) were stored in a freezer at -80 °C until further use.

### RNA extraction and quantitative real-time PCR

Total RNA was extracted from the GCF samples using a commercially available TaqMan miRNA Reverse Transcription kit. RNA quantity and purity were determined using a NanoDrop 2000 spectrophotometer (Thermo Fisher Scientific, Waltham, MA, USA). Reverse transcription was performed using the TaqMan® MicroRNA Reverse Transcription Kit to generate cDNA. Real-time PCR was carried out using the TaqMan® MicroRNA Assays Kit on Applied BioSystems 7900HT thermocycler (Applied Biosystems. Inc, CA; USA). The miRNA expression levels were quantified using the 2^−ΔΔCT^ method and normalized to the internal control hsa-miR-16-5p [12].

### Statistical analysis

Statistical analysis was performed using SPSS software version 23.0 (IBM, Armonk, NY, USA). The normality of the data distribution was assessed using the Kolmogorov-Smirnov test. The continuous data were presented as mean ± standard deviation or median ± interquartile range (IQR). The differences between the CP and the healthy control groups were analyzed using the student’s t-test or the Mann-Whitney U test, as appropriate. Fisher exact test was used for analyzing categorical data. The correlation between miRNA expression levels and clinical parameters was analyzed using Pearson’s correlation analysis. The receiver operating characteristic (ROC) curve analysis was performed to assess the diagnostic potential of miR-200 family members in CP. A *P* value of < 0.05 was considered statistically significant.

## Results

### Patient characteristics

As shown in Table 1, the study included 113 healthy individuals as controls, who were matched with 103 CP patients based on age and gender (both *P* > 0.05). Moreover, there were no significant differences in BMI, fasting glucose, smoking status, and ferritin between the CP patients and the control group (all *P* > 0.05). However, a higher level of hs-CRP was revealed in CP patients compared to healthy individuals (*P* < 0.001). In the CP group, some patients had lost up to 7 teeth due to the condition, while the median number of teeth lost was 0. The interquartile range (IQR) of 3 suggested that 75% of patients in the chronic periodontitis group had lost 3 or fewer teeth. On the other hand, none of the healthy controls showed any tooth loss due to periodontitis. Clinical parameters including PI, GI, BOP, CAL, and PPD were significantly higher in the CP group when compared to the control group (all *P* < 0.001).

**Table 1:** Demographic and clinical characteristics of study participants

Characteristics	Chronic periodontitis (n = 103)	Healthy controls (n = 113)	*P*
Age	46 (40 ~ 53)	45 (37 ~ 53)	0.530
Gender			
Male	50	62	
Female	53	51	0.414
Education level			
Primary school	43	44	
High school	40	47	
University	20	22	0.902
Body mass index (kg/m^2^)	20.9 (19.2 ~ 22.2)	21.1 (19.7 ~ 22.85)	0.123
Fasting glucose (mg/dL)	83.7 (80.6 ~ 87.1)	83.2 (80.05 ~ 86.1)	0.286
Smoking status			
Never smokers	90	91	
Past smokers	10	15	
Current smokers	3	7	0.342
Ferritin (ng/mL)	150.3 (92.3 ~ 216.8)	151.6 (98.0 ~ 219.1)	0.430
hs-CRP (mg/dL)	0.390 (0.190 ~ 0.620)	0.270 (0.155 ~ 0.375)	< 0.001
CAL (mm)	4.8 (3.6 ~ 5.8)	1.6 (0.7 ~ 2.4)	< 0.001
PPD (mm)	4.9 (2.9 ~ 6.8)	1.9 (1.3 ~ 2.45)	< 0.001
PI	1.5 (1.1 ~ 1.7)	0.5 (0.3 ~ 0.7)	< 0.001
GI	1.4 (1.1 ~ 1.7)	0.5 (0.3 ~ 0.75)	< 0.001
BOP (%)	19.3 (11.6 ~ 25.8)	4.9 (2.65 ~ 7.5)	< 0.001

Note: hs-CRP = high-sensitivity C-reactive protein, PI = plaque index, PD = Probing Pocket Depth, CAL = clinical attachment level, GI (Gingival Index), BOP (Bleeding on Probing, %). All continuous values were presented as median ± interquartile range (IQR)

### MiR-200 family expression levels in GCF

**Fig. 1:**
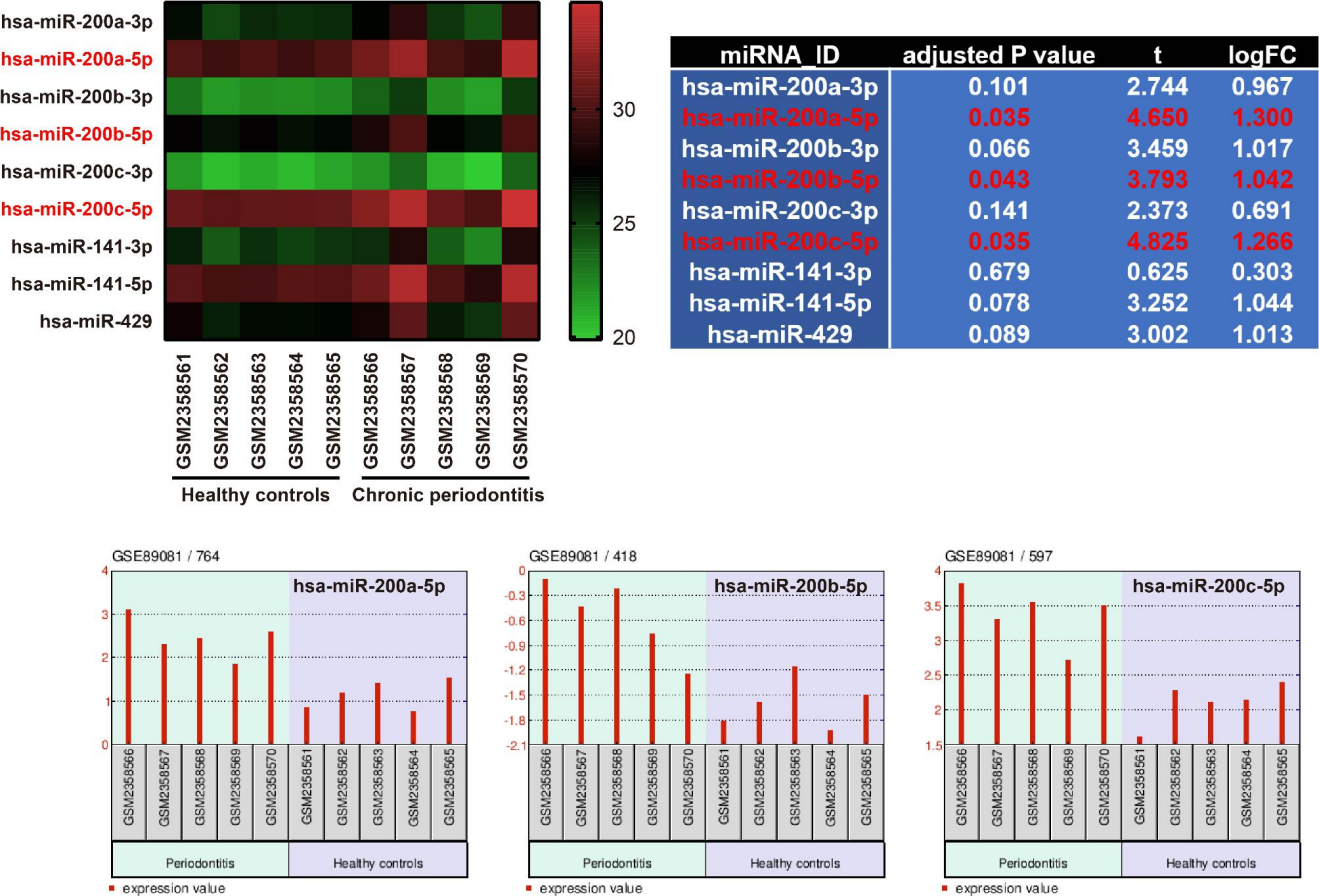
Comparison of miR-200 family expression levels in gingival crevicular fluid (GCF) of chronic periodontitis (CP) patients and healthy controls by analyzing the GSE89081 dataset using GEO2R

**Fig. 2:**
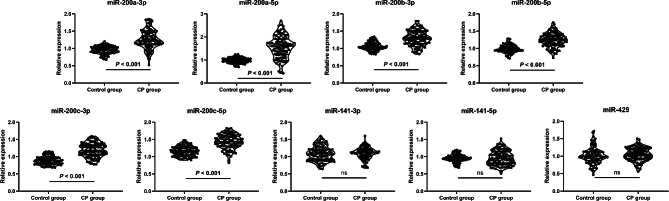
Quantitative real-time PCR was employed to assess the levels of miR-200 family expression in the gingival crevicular fluid (GCF) of both chronic periodontitis (CP) patients and healthy controls

By analyzing the GSE89081 dataset using GEO2R, it was found that miR-200a-5p, miR-200b-5p and miR-200c-5p were significantly upregulated in GCF of the CP patients compared to the healthy controls, with log2|fold change (FC)|> 1 and adjusted *P* < 0.05 (Fig. 1). In this study, the expression levels of miR-200a-3p, miR-200a-5p, miR-200b-3p, miR-200b-5p, miR-200c-3p and miR-200c-5p were validated to be significantly higher in the GCF of CP patients than the healthy controls (*P* < 0.05), as demonstrated in Fig. 2. However, no significant difference was found in the expression levels of miR-141-3p, miR-141-5p, and miR-429 between the two groups (*P* > 0.05).

### Diagnostic value of miR-200a, miR-200b, and miR-200c expressions in GCF for CP

**Fig. 3:**
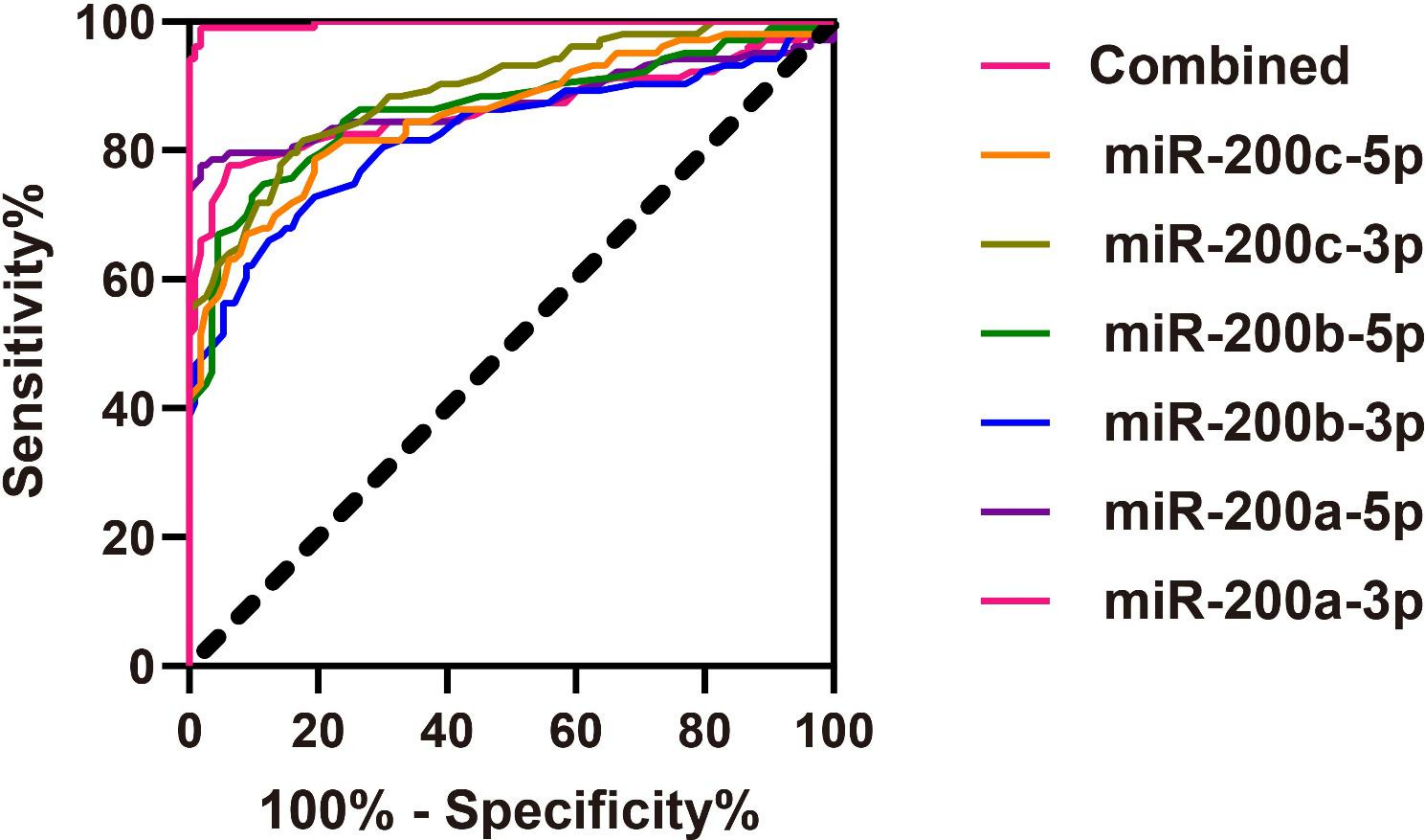
Diagnostic value of miR-200a, miR-200b, and miR-200c expressions in gingival crevicular fluid (GCF) for chronic periodontitis (CP)

As demonstrated in Table 2, three miRNAs (miR-141-3p, miR-141-5p, miR-200b-3p) in GCF were found to have no diagnostic value for CP, with sensitivities ranging from 25.24 to 81.55%, specificities ranging from 34.51 to 94.69%, and AUC values ranging from 0.508 to 0.567. However, miR-200a-5p showed the highest diagnostic performance with an AUC of 0.876, sensitivity of 78.64%, and specificity of 97.35%. Other miRNAs, including miR-200a-3p, miR-200b-3p, miR-200b-5p, miR-200c-3p, and miR-200c-5p, also demonstrated good diagnostic value with AUC values ranging from 0.832 to 0.893. When the six miRNAs (miR-200a-3p, miR-200a-5p, miR-200b-3p, miR-200b-5p, miR-200c-3p, and miR-200c-5p) were combined, they demonstrated excellent diagnostic value with an AUC of 0.997, sensitivity of 99.03%, and specificity of 98.23% (Fig. 3). These findings suggest that these miRNAs in GCF may be useful biomarkers for the diagnosis of CP.

**Table 2:** Diagnostic value of miR-200 family members in gingival crevicular fluid (GCF) for CP (CP)

miRNAs	Cut-off	Sensitivity	specificity	Youden Index	AUC	95%CI	*P*
miR-200a-3p	1.102	77.67%	93.81%	0.715	0.865	0.811 ~ 0.920	1.82E-20
miR-200a-5p	1.192	78.64%	97.35%	0.760	0.876	0.822 ~ 0.930	1.35E-21
miR-200b-3p	1.213	67.96%	87.61%	0.556	0.832	0.775 ~ 0.889	3.58E-17
miR-200b-5p	1.124	74.76%	88.50%	0.633	0.864	0.813 ~ 0.916	2.44E-20
miR-200c-3p	1.002	81.55%	83.19%	0.647	0.893	0.851 ~ 0.935	2.06E-23
miR-200c-5p	1.279	79.61%	79.65%	0.593	0.857	0.805 ~ 0.908	1.48E-19
miR-141-3p	0.963	81.55%	34.51%	0.161	0.537	0.459 ~ 0.614	3.51E-01
miR-141-5p	1.135	25.24%	94.69%	0.199	0.508	0.426 ~ 0.591	8.32E-01
miR-429	1.137	35.92%	79.65%	0.156	0.567	0.491 ~ 0.644	8.79E-02

### Correlation between miR-200a, -200b and -200c expressions in GCF and clinical parameters of CP

**Fig. 4:**
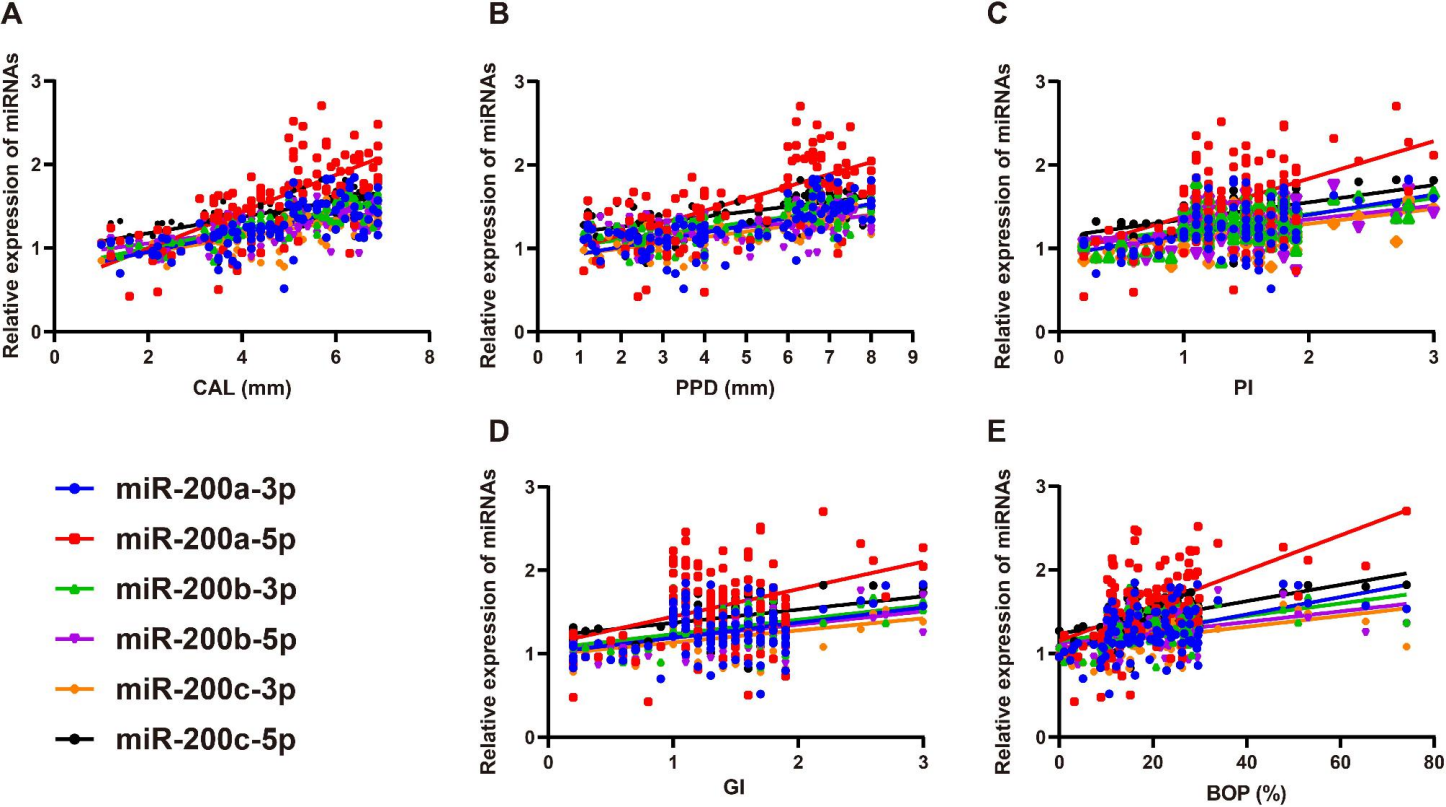
Pearson correlation analysis between miR-200a, -200b and -200c expressions in gingival crevicular fluid (GCF) and clinical parameters of chronic periodontitis (CP) Note: Correlation between miR-200a, -200b, and -200c expressions in GCF and various clinical parameters of CP, including clinical attachment level (CAL, A), probing pocket depth (PPD, B), plaque index (PI, C), gingival index (GI, D), and bleeding on probing (BOP, E)

Table 3 showed the correlation coefficients between miRNA expression levels and various parameters related to periodontal disease, including CAL, PPD, PI, and BOP. MiR-141-3p, miR-141-5p, and miR-429 exhibited weak correlations with these parameters, suggesting that their expression levels may not be closely related to CP progression (all *P* > 0.05). However, miR-200a-3p, miR-200a-5p, miR-200b-3p, miR-200b-5p, miR-200c-3p, and miR-200c-5p showed significant and positive correlations with CAL, PPD, PI, and BOP (all P < 0.05, as shown in Fig. 4), indicating that their expression levels may be associated with the severity of CP.

**Table 3:** Correlation between miR-200 family members in gingival crevicular fluid and clinical parameters of chronic periodontitis

miRNAs		CAL (mm)	PPD (mm)	PI	GI	BOP (%)
miR-200a-3p	r	0.718	0.666	0.408	0.230	0.429
	*P*	**< 0.001**	**< 0.001**	**< 0.001**	**0.019**	**< 0.001**
miR-200a-5p	r	0.742	0.652	0.401	0.205	0.504
	*P*	**< 0.001**	**< 0.001**	**< 0.001**	**0.038**	**< 0.001**
miR-200b-3p	r	0.836	0.664	0.363	0.278	0.443
	*P*	**< 0.001**	**< 0.001**	**< 0.001**	**0.004**	**< 0.001**
miR-200b-5p	r	0.550	0.475	0.350	0.316	0.400
	*P*	**< 0.001**	**< 0.001**	**< 0.001**	**0.001**	**< 0.001**
miR-200c-3p	r	0.734	0.677	0.395	0.246	0.399
	*P*	**< 0.001**	**< 0.001**	**< 0.001**	**0.012**	**< 0.001**
miR-200c-5p	r	0.728	0.547	0.410	0.266	0.506
	*P*	**< 0.001**	**< 0.001**	**< 0.001**	**0.007**	**< 0.001**
miR-141-3p	r	0.141	0.074	0.110	0.050	-0.098
	*P*	0.156	0.459	0.268	0.614	0.326
miR-141-5p	r	0.019	0.013	0.078	0.034	-0.013
	*P*	0.846	0.900	0.436	0.735	0.896
miR-429-5p	r	0.085	0.134	-0.087	-0.064	-0.042
	*P*	0.395	0.178	0.382	0.524	0.671

Note: Clinical attachment level (CAL); Probing pocket depth (PPD); Plaque index (PI); Gingival index (GI); Bleeding on probing (BOP).

## Discussion

CP is a prevalent inflammatory disease that affects the supporting tissues of teeth, leading to tooth loss and a significant impact on patients’ quality of life [25, 26]. Dysregulation of miRNA expression has been associated with various pathologies, making them excellent molecules to study as potential biomarkers for CP [19, 27]. While initial studies focused on gingival tissue, there has been a recent shift in interest towards GCF and saliva as potential sources of miRNAs [28]. GCF, as an inflammatory exudate originating from gingival microcirculation and crossing the inflamed periodontal tissues, has been proposed as an excellent research source for miRNA biomarkers due to its ease of isolation and identification through qPCR, less invasive sampling, and the high stability of miRNAs commonly used in analytical assays [29, 30]. These factors indicate that GCF is a valuable oral biofluid for distinguishing between periodontal health and disease status.

The miR-200 family consists of two gene clusters: miR-200a/miR-200b/miR-429 located on human chromosome 1p36.33 and miR-200c/miR-141 on chromosome 12p13.31. This family has been found to regulate various genes responsible for different dental epithelial cell lineages [31, 32]. In the context of miRNA studies and periodontitis, machine learning algorithms can process large amounts of miRNA expression data from diverse sources, such as GCF samples, to identify specific miRNA signatures associated with the development and progression of periodontitis [19, 33]. In present study, by analyzing the GSE89081 dataset using GEO2R, it was found that miR-200a-5p, miR-200b-5p and miR-200c-5p were significantly upregulated in GCF of the CP patients compared to the healthy controls. Expression levels of miR-200a, -200b and -200c were validated to be significantly increased in GCF of CP patients compared to the healthy control, while miR-141 and miR-429 did not show significant differences. The results of ROC demonstrated that miR-200a, -200b and -200c expressions in GCF demonstrated good diagnostic value with AUC values ranging from 0.832 to 0.893. When the six miRNAs (miR-200a-3p, miR-200a-5p, miR-200b-3p, miR-200b-5p, miR-200c-3p, and miR-200c-5p) were combined, they demonstrated excellent diagnostic value with an AUC of 0.997, sensitivity of 99.03%, and specificity of 98.23%. These findings suggest that these miRNAs in GCF may be useful biomarkers for the diagnosis of CP. Besides, miR-200a, -200b and -200c expressions in GCF showed significant and positive correlations with CAL, PPD, PI, and BOP, indicating that their expression levels may be associated with the severity of CP.

MiR-200a was dysregulated in several inflammatory diseases. For example, in human hepatocytes treated with free fatty acids and inflammatory factors, miR‐200a levels were increased [34]. MiR-200a-5p was significantly upregulated in liver biopsies of hepatitis C patients with advanced fibrosis compared to those with early fibrosis [35]. Worth mentioning, miR-200a-3p in saliva was upregulated in the patients with progressive CP compared to the control group, demonstrating good diagnostic value with an AUC value of 0.686 [36], which is consistent with this study.

MiR-200b is associated with inflammatory diseases such as periodontitis. The relative levels of miR-200b in GCF and gingival tissues were significantly higher in CP patients and positively correlated with tumor necrosis factor-alpha [29, 37]. MiR-200b levels were also significantly elevated in the gingiva of obese periodontitis subjects [38]. Moreover, GCF miR-200b-3p and miR-200b-5p were higher in subjects with periodontitis and periodontitis + cardiovascular disease compared to healthy controls and subjects with cardiovascular disease alone [19]. Inflammation cytokines such as IL-1β, IL-6, and TNF-α induced miR-200b expression in human gingival fibroblasts (HGF), and miR-200b attenuated the production of inflammatory cytokines such as IL-6 and IL-1β through a negative feedback loop with the NF-κB pathway in the inflamed gingiva [39]. Microarray analysis in a Japanese study showed increased miRNA-200b levels in inflamed gums and reduced levels in healthy gums [37]. Consistent with the results of this study, Elazazy O et al. reported that miR-200b was positively correlated with CAL, PPD, and TNF-α levels in CP patients [29].

MiR-200c directly targets 3′ UTRs of IL-6, IL-8, interferon-related developmental regulator 1 (Ifrd1), and chemokine (C-C motif) ligand 5 (CCL-5), and downregulated their expression in human periodontal ligament, gingival fibroblasts, and the periodontium of periodontitis rats [40]. MiR-200c-3p has been reported to exert an anti-inflammatory role in pre-osteoblasts and HGFs, attenuating the development of periodontitis [40, 41]. Plasma-derived exosomal miR-200c-3p has also been suggested as a valuable biomarker for periodontitis [42]. However, a previous study reported significantly reduced miR-200c expression in gingival tissues of periodontitis patients [43]. In contrast, significantly increased expression levels of miR-200c were found in GCF of CP patients compared to healthy controls. This discrepancy may be attributed to different sample sources, with the previous study using gingival tissues and this study using GCF.

The miR-200 family members have been shown to regulate innate immune response by targeting the TLR4 signaling pathway via MyD88, which is essential for mediating signals through TLR [44]. TLRs played a significant role in initiating early periodontitis and promoting its advancement, and the levels of TLR4 expression exhibit a marked increase in gingivitis and various periodontal tissues including pocket epithelium, spinous epithelial layer, gingival fibroblasts, periodontal ligament fibroblasts, and connective tissues [45, 46]. Thus, I speculate that the increased expression of miR-200a, miR-200b, and miR-200c in GCF during an inflammatory state may inhibit the TLR4 signaling pathway, thereby attenuating the progression of CP. Further exploration is needed to confirm this hypothesis.

Limitations should be acknowledged when interpreting the results of this study. Firstly, the expression levels of miR-200a, miR-200b, and miR-200c in GCF showed significant differences between CP patients and healthy controls, but there was considerable overlap in the expression ranges between the two groups. This suggests that miR-200 alone may not be sufficient for a definitive diagnosis of CP. Secondly, the observed magnitude of difference in expression levels for miR-200 between the CP and control groups was relatively small, with the highest expression value in the CP group being less than twice that of the control group. While this indicates a relative increase in expression, it may not be substantial enough to serve as a standalone diagnostic indicator. It is important to consider that individual variations and the influence of confounding variables may contribute to the observed expression levels. Additionally, this study focused solely on the expression of miRNAs in GCF and did not consider other potential biomarkers or factors that could play a role in the development or progression of CP. It would be valuable for future research to explore additional biomarkers and factors associated with CP to provide a more comprehensive understanding of the disease. Furthermore, the study did not include a control group with a different periodontal disease than CP. As a result, it may be challenging to differentiate between miRNA expression specific to CP and miRNA expression associated with more general periodontal diseases.

## Conclusion

MiR-200a, miR-200b, and miR-200c demonstrated significant upregulation in the GCF of CP patients compared to healthy controls. These miRNAs showed good diagnostic value and exhibited positive correlations with clinical parameters of CP severity. However, it is important to consider the limitations of this study, such as the overlap in expression ranges, the relatively small magnitude of difference in expression levels, the need to explore other biomarkers and factors associated with CP, and the distinction between CP-specific miRNA expression and more general periodontal disease-related expression. Future investigations should address these limitations and further explore the functional implications of miR-200 as a potential therapeutic target or prognostic indicator for periodontal disease progression.

## Data Availability

The datasets used and/or analyzed during the current study are available from the corresponding author on reasonable request.
